# The impact of social presence on perceived learning performance in online education

**DOI:** 10.3389/fpsyg.2026.1841861

**Published:** 2026-08-12

**Authors:** Lu Yang, Qiongbin Yin

**Affiliations:** 1 Department of Management, College of Economics and Management, Nanjing Agricultural University, Nanjing, Jiangsu, China; 2 School of Business, Nanjing University, Nanjing, Jiangsu, China

**Keywords:** individualism, intrinsic motivation, online education, perceived learning performance, social presence

## Abstract

Building on digital information technology, online education in Chinese universities has developed rapidly. While it offers flexibility, it often reduces student engagement and increases learning burnout. How to fully leverage the advantages of online education while mitigating its negative effects has become a key concern in online learning research. The current study investigates how social presence influences undergraduates’ perceived learning performance in online teaching, focusing on the mediating role of learning burnout and the moderating effects of individualism and intrinsic learning motivation. A survey was conducted with 611 university students in China, and data were analyzed using structural equation modeling and PROCESS regression. Results showed that: (1) social presence in online courses significantly enhances perceived learning performance; (2) learning burnout mediates this relationship; (3) individualism and intrinsic learning motivation moderate this effect. Specifically, the indirect effect of social presence on perceived learning performance through reduced learning burnout is stronger among students with lower individualism or higher intrinsic motivation. These findings offer practical implications for improving teaching effectiveness, enhancing learning outcomes, and alleviating learning burnout in online education.

## 1 Introduction

Due to the COVID-19 pandemic and the rapid advancement of digital information technologies, Chinese universities have rapidly expanded the adoption of online and blended teaching (Yang and Lay, 2025). Online education, supported by internet infrastructure and digital technologies, refers to a mode of teaching and learning conducted partially or entirely online. It enables students to access educational content and interact with teachers and peers beyond the constraints of time and location (Sun and Chen, 2016). Recent research has examined online education either from the view of technology accepatance, showing that students’ performance depends on perceived usefulness, digital skills, and institutional support (Younas et al., 2026), or from the perspective of personalization, suggesting that online education should be tailored to meet the diverse needs of students (Najjar et al., 2025). More specifically, Hsu et al. (2025) conducted a systematic review and identified several factors influencing the acceptance of online education, including technology design, individual characteristics, and social factors.

Compared with traditional face-to-face instruction, online education offers both substantial advantages and notable challenges. On the one hand, it provides greater flexibility, supports diverse instructional methods, and demonstrates resilience in responding to public health crises such as COVID-19. On the other hand, it is often criticized for weakened instructional quality control, increased psychological distance between teachers and students, and decreased classroom engagement, which may result in heightened learning burnout (i.e., the gradual onset of exhaustion, cynicism, and loss of commitment; Maslach and Jackson, 1981) and lower learning performance. As such, how to capitalize on the advantages of online education while mitigating its drawbacks has become a central focus of online learning research.

Given the dual roles of teachers and students in the teaching–learning process, prior studies have examined strategies to reduce students’ learning burnout from both perspectives. For example, Kim et al. (2018), in a meta-analysis, emphasized that support from schools or teachers is particularly effective in alleviating student learning burnout. From the student perspective, Barratt and Duran (2021) demonstrated that psychological capital (i.e., self-efficacy, optimism, hope, and resilience) can help reduce burnout and enhance engagement in online distance learning. Building on self-determination theory and the Stimuli-Organism-Response framework, Tan et al. (2025) found that mindfulness can fulfill students’ psychological needs, such as structural and social bonds, and reduce learning burnout. Taking a more comprehensive approach, Aria et al. (2024) employed grey Delphi and grey-DEMATEL techniques to investigate the factors influencing student burnout in online education during the COVID-19 pandemic. Among the seven identified factors, technology infrastructure emerged as the most influential. Other cause-related factors, in descending order of impact, include institutional facilities and professor characteristics. The effect-related factors comprise the educational environment and course structure, student characteristics, social–emotional factors, and the family/home environment.

Therefore, beyond the roles of teachers and students, online platforms also play a critical role in shaping online learning experiences. Moreno et al. (2017) showed that students’ perceptions of a platform’s usefulness and ease of use positively influence their intention to engage with e-learning systems, thereby improving learning performance. Moreover, the dynamic interactions among teachers, students, and platforms contribute to the development of a supportive online learning environment. In this regard, Kreijns et al. (2013) proposed the SIPS model, which includes four key components: sociability, social interaction, social presence, and social space. Specifically, sociability reflects the platform’s capacity to support social interaction and social space; social interaction pertains to the frequency and quality of communication among participants; social presence is defined as the extent to which individuals are perceived as real people; and social space refers to the perceived sense of community and emotional connection among participants. Within this framework, sociability facilitates social interaction, which in turn enhances both social presence and social space. Notably, social presence is also believed to directly contribute to the development of social space.

Given the conceptual overlaps among these constructs, the current study focuses specifically on the role of *social presence* in online education. Drawing on previous literature, we define social presence as the degree to which students perceive the teacher as a real presence in the online learning environment. While the concept of social presence has been extensively studied in domains such as livestreaming commerce, particularly in relation to how live-streamers’ behaviors and characteristics influence consumers’ perceptions of social presence (e.g., Lin and Lee, 2024; Gao et al., 2023), recent research in the context of online education has primarily focused on identifying factors that enhance social presence, its moderating effects, and its influence on student satisfaction. However, there is a lack of empirical evidence regarding whether and how social presence affects university students’ learning performance in online courses. To address these gaps, the present study investigates the effect of social presence on undergraduates’ perceived learning performance in online education and further explores the underlying mechanism and boundary conditions. Specifically, we examine the mediating role of learning burnout and explore whether individual differences—namely, individualism and intrinsic learning motivation—moderate the proposed relationships. The findings aim to enrich the theoretical understanding of online learning processes and provide actionable insights for improving the effectiveness of online teaching in higher education.

### 1.1 Social presence and perceived learning performance

Social presence refers to the extent to which individuals are perceived as a real presence and experience a sense of connectedness with others in mediated communication (Short et al., 1976). Higher levels of social presence are thought to reduce psychological distance and facilitate the development of interpersonal relationships. In educational contexts, it is typically defined as the extent to which students perceive the presence of others—actual or potential—during the learning process. This perception can be viewed from two perspectives: teachers’ perception of students’ presence and students’ perception of teachers’ presence (Wang et al., 2021). The present study adopts the latter perspective, emphasizing a key challenge in online education: the psychological and communicative distance caused by the virtual environment. Reduced cues and the lack of physical proximity may weaken students’ subjective perceptions of the teacher’s presence, hindering the formation of a positive teacher-student relationship (Weidlich et al., 2024). In other words, teachers in online classes may appear more like virtual agents than human agents, making it difficult for students to maintain attention and increasing the likelihood of fatigue. These effects collectively diminish students’ sense of immersion and identification with the learning experience—namely, low social presence. Therefore, the current work focuses on students’ perceived social presence of teachers in online education and its subsequent influence on learning performance.

Learning performance does not have a universally accepted definition. The Association for Educational Communications and Technology (AECT) defines it as learners’ ability to apply newly acquired knowledge and skills, encompassing both mastery and flexible application. Previous research suggests that learning performance comprises two dimensions: learning efficiency—achieving greater output with less input—and learning achievement, which includes both the attainment of learning goals and the subjective experience of satisfaction or a sense of accomplishment (Lindsay, 1982; Omar et al., 2015). Building on this, recent research has started to examine students’ perceived learning performance in online contexts and its antecedents. For example, Chen and Tsai (2025) demonstrated that the use of interactive e-books can increase students’ academic performance, engagement, satisfaction, and perceived learning outcomes. Similarly, Wang and Antonenko (2017) found that instructor presence in teaching videos (vs. absence) improved students’ visual attention, information recall, and perceived learning and satisfaction. In the current work, we adopt the construct of perceived learning performance, which refers to students’ self-assessment of the knowledge and skills acquired through learning (Wan et al., 2008). This definition aligns with our goal of capturing students’ subjective evaluations of their learning outcomes in online courses.

Drawing on the literature on social presence and learning performance, we argue that social presence in online education plays a crucial role in shaping students’ perceived learning performance. A high level of social presence can reduce the psychological distance between teachers and students (Weidlich et al., 2024), thereby enhancing students’ perception of the teacher as a supportive individual. This perception may in turn increase perceived teacher social support (Wang, 2025), student engagement (Miao et al., 2022), stimulate learning motivation (Hoogerheide et al., 2016), and elevate students’ arousal and attention levels (Zhang et al., 2022)—all of which are factors that reduce learning burnout and enhance perceived learning performance. Moreover, evidence from related domains also supports this argument. For instance, in marketing research, Wang et al. (2025) found that in live streaming commerce, social presence evoked by highly interactive streamers led consumers to experience the immpersive state of flow—a deep, engaging experience during which individuals lose awareness of time and fatigue—which ultimately increased their purchase intention. Similarly, we propose that social presence in online education can immerse students in the learning experience and enhance their perceived learning outcomes. Formally:

*H1*: Social presence in online courses has a significant positive effect on students’ perceived learning performance.

### 1.2 The mediating role of learning burnout

Learning burnout is defined as a negative psychological state wherein learners, influenced by internal traits or external situational factors, perceive heightened academic stress that leads to disengagement or avoidance behaviors (Tang et al., 2021). As mentioned earlier, in online education, social presence has been identified as a key factor in alleviating learning burnout through several pathways: perceived teacher social support, increased student engagement, strengthened learning motivation, and elevated arousal levels. Regarding *perceived social support*, a high level of social presence allows students to perceive the instructor as a supportive individual, thereby enhancing their sense of receiving instrumental, emotional, and informational support. This perceived support has been shown to mitigate feelings of burnout (Romano et al., 2021; Singh and Ishrat, 2025). In terms of s*tudent engagement*, social presence fosters a positive classroom atmosphere and reduces the psychological distance between students and teachers, which in turn increases student engagement and participation. For instance, Miao et al. (2022) found that social presence significantly enhances students’ learning engagement in online environments. With respect to *learning motivation*, when students perceive a strong social presence, they are more likely to invest effort in cognitive processing and learning activities, thereby reducing feelings of fatigue and frustration (Hoogerheide et al., 2016). Finally, in terms of *arousal levels*, social presence can heighten students’ arousal levels, enhancing working memory and information retention, which contributes to a sense of achievement and helps reduce burnout (Hoogerheide et al., 2019).

Prior literature on the consequences of learning burnout has demonstrated that it leads to a range of negative emotional and behavioral outcomes, including negative feelings, lower self-efficacy, and reduced academic performance. For example, Neumann et al. (1990) found that learning burnout led to students’ emotional exhaustion. Similarly, Wei et al. (2021) showed that student learning burnout harms students’ self-concept and engagement, negatively affecting their health and academics. More directly, Madigan and Curran (2021) conducted a meta-analysis on the relationship between burnout and academic achievement and demonstrated that burnout reduces self-efficacy and academic achievement across school, college, and university settings.

This issue is particularly important in online learning environments, as the physical separation between students and teachers often weakens emotional expression and immediate feedback, thereby reducing students’ perceived social support, engagement, learning motivation, and arousal levels. These factors negatively affect students, making them more prone to negative psychological experiences such as emotional exhaustion and a diminished sense of achievement (Neumann et al., 1990; Wei et al., 2021). Students may also exhibit adverse learning behaviors such as mind-wandering during class, low enthusiasm for learning, and reduced post-class effort, all of which contribute to learning burnout. In contrast, enhancing social presence in online courses can improve students’ perception of the instructor’s real presence and foster a positive learning atmosphere. This, in turn, stimulates students’ positive attitudes and behaviors and enhances their learning performance. These findings are consistent with research in traditional classroom settings (Watt et al., 2017), which suggests that a positive classroom climate helps alleviate student learning burnout. Therefore, based on the negative relationship between social presence and learning burnout, as well as the negative impact of learning burnout on perceived learning performance, this research proposes that social presence in online courses can enhance perceived learning performance by reducing learning burnout.

*H2*: Learning burnout mediates the relationship between social presence and students’ perceived learning performance.

However, the mediating effect of learning burnout may be moderated by students’ individual traits or external environmental factors. This study focuses on the former, examining whether individualism and intrinsic learning motivation moderate this indirect effect.

### 1.3 The moderating role of students’ individualism

The concept of individualism originates from cross-cultural psychology and has since been widely applied in research across consumer behavior and organizational studies to describe individuals’ cultural orientations and their corresponding psychological and behavioral characteristics. Individualism is commonly associated with Western cultures, which emphasize independence and the uniqueness of the self, whereas collectivism is more prevalent in Eastern cultures, emphasizing interdependence and loyalty to the group. Importantly, individualism and collectivism are not mutually exclusive; they can coexist within the same cultural context. This duality enables the exploration of individual-level variations in cultural orientation within a single cultural setting. According to Markus and Kitayama (1991), individualism is linked to an independent self-construal, wherein individuals tend to perceive themselves as autonomous entities and prioritize personal thoughts and goals. In contrast, collectivism is associated with an interdependent self-construal, in which individuals define themselves in relation to others and demonstrate a greater degree of social dependency. Building on this conceptual framework, Teoh et al. (1999) found that, compared to students from individualistic cultures, those from collectivistic cultures tended to perceive a lower degree of risk in questionable actions, particularly when such actions would benefit their close or moderately close in-groups.

Individualism also influences individuals’ attentional orientation. Those with high levels of individualism are more likely to focus on objects and task-related information, whereas individuals with lower levels of individualism tend to be more sensitive to contextual and environmental cues (Markus and Kitayama, 1991). For instance, Greenfield et al. (2006) found that high-individualism cultures tend to value scientific or cognitive forms of intelligence, thereby promoting active student participation. In contrast, low-individualism cultures place greater emphasis on social or relational intelligence, with students relying more heavily on interaction and collaboration with teachers and peers. These cultural differences have important implications for online education. Students with high individualism may concentrate more on course content, while those with low individualism may be more influenced by contextual factors such as the presence or absence of the teacher, commonly referred to as social presence. When social presence is perceived to be high, it can reduce students’ learning burnout and, in turn, enhance their perceived learning performance. For example, Chen et al. (2019) compared student evaluations across cultural contexts and found that Chinese students (from a lower-individualism culture) reported closer relationships and fewer conflicts with teachers than their Dutch counterparts (from a higher-individualism culture).

Building on this literature, we propose that students with lower levels of individualism are more susceptible to the effect of social presence on learning burnout. In other words, the effect of social presence in reducing learning burnout is expected to be stronger among students with lower levels of individualism. Therefore, we hypothesize that individualism negatively moderates both the relationship between social presence and learning burnout and the indirect effect of social presence on perceived learning performance via learning burnout.

*H3*: Students’ individualism negatively moderates the relationship between social presence and learning burnout.

*H4*: The indirect effect of social presence on perceived learning performance through learning burnout is moderated by individualism.

### 1.4 The moderating role of students’ intrinsic motivation

Motivation has been a central construct in both psychology and educational research. Learning motivation, in particular, is regarded as a key psychological driver that initiates, sustains, and directs individuals’ engagement in learning activities toward the achievement of specific educational goals. Prior literature typically categorizes learning motivation into two primary types: intrinsic motivation and extrinsic motivation. Intrinsic motivation refers to engaging in learning for its inherent value, such as curiosity, interest, or a sense of personal fulfillment. In contrast, extrinsic motivation is driven by external factors unrelated to the learning activity itself, such as the desire to obtain rewards, avoid punishment, or achieve good grades (Ryan and Deci, 2000; Yildirim et al., 2024). Extensive empirical research has demonstrated that intrinsic motivation positively influences student engagement (Ali et al., 2022), reduces feelings of loneliness (Yildirim et al., 2024), and contributes to improved learning performance (Mekler et al., 2017). It is worth noting that intrinsic motivation is particularly crucial in online educational environments, where students often study in isolation and lack the extrinsic motivators present in face-to-face learning settings (Fırat et al., 2018). Moreover, Cho and Heron (2015) explored the role of learning motivation in online education and found that intrinsic motivation enhances online learning engagement, whereas extrinsic motivation may hinder it by increasing avoidance or disengagement from learning activities.

Collectively, these findings suggest that intrinsic motivation is positively associated with students’ proactive learning behaviors. Students with higher levels of intrinsic motivation are more likely to independently seek out, access, and utilize learning resources to support improved learning performance. In online learning environments, such students are also more attuned to factors that enhance learning efficiency, such as social presence. Specifically, students with high intrinsic motivation are more likely to value elements that foster learning engagement and involvement (Xiong et al., 2015), which in turn help them mitigate learning burnout (Pap et al., 2021) and enhance perceived learning performance. For example, Pap et al. (2021) found that teacher support increases students’ intrinsic motivation, which positively predicts their study engagement and reduces both boredom and burnout. Based on this reasoning, we propose that students with higher levels of intrinsic motivation are more strongly influenced by the effect of social presence in reducing learning burnout through increased learning engagement. In other words, intrinsic motivation is expected to strengthen both the direct relationship between social presence and learning burnout and the indirect effect of social presence on perceived learning performance via learning burnout. Formally:

*H5*: Students’ intrinsic motivation positively moderates the relationship between social presence and learning burnout.

*H6*: The indirect effect of social presence on perceived learning performance through learning burnout is moderated by intrinsic motivation.

**Figure 1:**
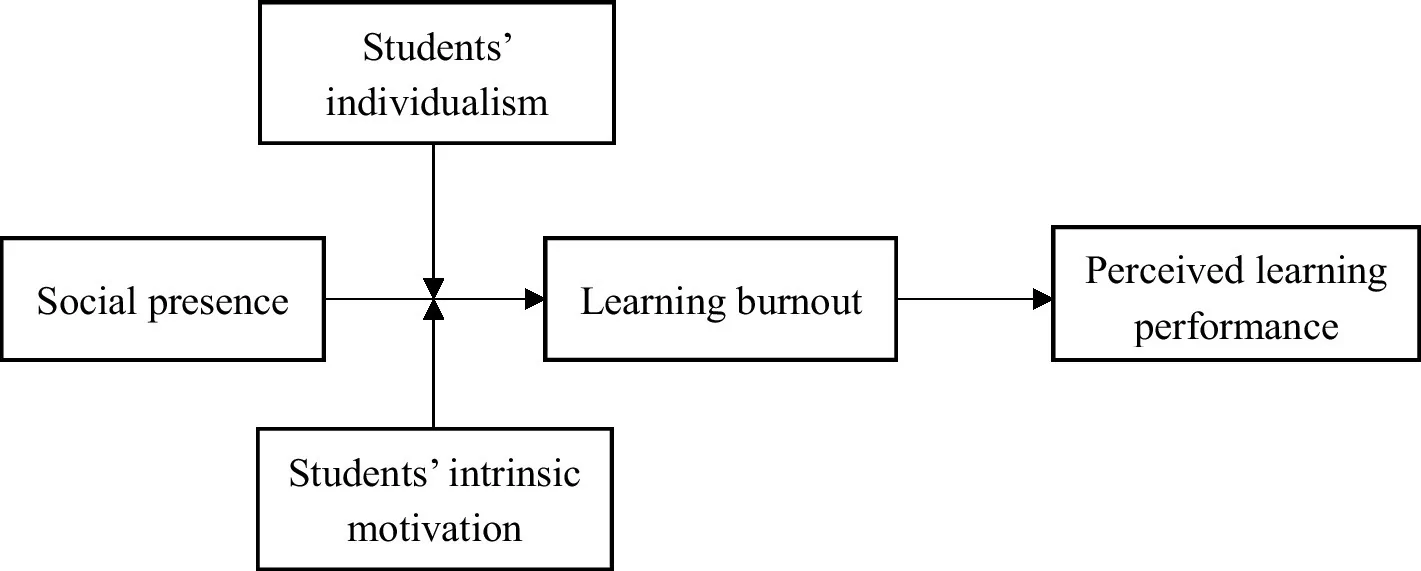
Theoretical framework.

In summary, this research integrates the concept of social presence from the marketing literature with the psychological theories of individualism and intrinsic motivation. By constructing a moderated mediation model, we examine how social presence (independent variable) influences perceived learning performance (dependent variable) through learning burnout (mediator), with students’ individualism and intrinsic motivation serving as moderators. The proposed theoretical framework is presented in Figure 1.

## 2 Methods

### 2.1 Sample characteristics and data collection

This study employed a convenience sampling method and recruited participants through the Credamo online research platform. A total of 611 Chinese undergraduate students were randomly selected. To ensure research ethics, all participants were informed that the survey was anonymous and that their data would be used exclusively for research purposes. Informed consent was obtained from all participants. After excluding responses from individuals who had never participated in online courses, 600 valid questionnaires were retained, yielding a valid response rate of 98.20%. Among the participants, 163 were male (27.2%) and 437 were female (72.8%), with an average age of 21.05 years (SD = 1.64). Participants were drawn from all academic years: 30 freshmen (5.0%), 165 sophomores (27.5%), 228 juniors (38.0%), and 177 seniors (29.5%). In terms of academic majors, 243 participants (40.5%) were from economics and management disciplines, 69 (11.5%) from humanities (literature, history, and philosophy), 49 (8.2%) from science, 130 (21.7%) from engineering, 56 (9.3%) from agriculture and medicine, 15 (2.5%) from law, 11 (1.8%) from arts, and 27 (4.5%) from education-related disciplines. It should be noted that to enhance the reliability of the findings, gender, age, academic year, and academic major were included as control variables. The results remained consistent. Please refer to Table 1 for the participants’ demographic information.

**Table 1:** Participants’ demographic information.

Characteristic	Frequency	Percentage
Gender		
Male	163	27.2%
Female	437	72.8%
Academic year		
Freshman	30	5.0%
Sophomore	165	27.5%
Junior	228	38.0%
Senior	177	29.5%
Academic majors		
Economics and management	243	40.5%
Humanities (literature, history, and philosophy)	69	11.5%
Science	49	8.2%
Engineering	130	21.7%
Agriculture and medicine	56	9.3%
Law	15	2.5%
Arts	11	1.8%
Education	27	4.5%

### 2.2 Measures

All constructs in this study were measured using established scales adapted from prior research (please see Table 2), with items rated on a 7-point Likert scale (1 = strongly disagree to 7 = strongly agree).

**Table 2:** Items by construct and reference source.

Construct	Dimension	Number of items	Reference
Social presence		4	Adapted from Ogara et al. (2014) and Park and Kim (2020)
Learning burnout	Emotional exhaustion	8	Adapted from Maslach and Jackson (1981) and Wu et al. (2010)
	Inappropriate learning behavior	6	
	Reduced sense of accomplishment	6	
Perceived learning performance	Perceived skill development	6	Adapted from Alavi (1994)
	Self-reported learning	3	
	Learning interest	3	
Individualism	Individualism score	9	Adapted from Chen et al. (2015)
	Collectivism score	9	
Intrinsic motivation		6	Adapted from Fırat et al. (2018) and Lin and Wang (2021)

#### 2.2.1 Social presence

Social presence in online education was assessed using a scale adapted from Ogara et al. (2014) and Park and Kim (2020), revised to fit the context of Chinese online courses. The scale measured students’ perceived social presence of teachers and included 4 items, such as “I feel as if I am with the teacher,” “I can easily sense the teacher’s presence,” “It feels like I am communicating face-to-face with the teacher,” and “I feel a close connection with the teacher.” Higher scores indicate greater perceived social presence. The adapted scale demonstrated good reliability in the current study (Cronbach’s *α* = 0.890).

#### 2.2.2 Learning burnout

Learning burnout was assessed using a scale adapted from Maslach and Jackson (1981) and Wu et al. (2010) to suit educational contexts. The original scale, developed for human services professionals, comprised three dimensions: emotional exhaustion, depersonalization, and reduced personal accomplishment. However, since depersonalization in workplace contexts reflects a dehumanized or detached attitude toward others, it is less applicable to educational contexts. In the setting of online education in China, this dimension was adapted to reflect inappropriate learning behaviors. Accordingly, the revised scale includes 20 items across three dimensions: *emotional exhaustion* (e.g., “I feel bored with studying”), *inappropriate learning behavior* (e.g., “I find myself lacking patience in learning”), and *reduced sense of accomplishment* (e.g., “I am competent in this course” [reverse coded]). Higher scores indicate greater levels of learning burnout. The Cronbach’s alpha coefficients for the three subscales were 0.830, 0.893, and 0.818, respectively, with an overall reliability of 0.935 for the adapted scale.

#### 2.2.3 Perceived learning performance

Perceived learning performance was assessed using a scale adapted from Alavi (1994), consisting of 12 items across three subscales: *perceived skill development*, *self-reported learning*, and *learning interest*. Example items include: “My ability to analyze issues has improved,” “My understanding of basic concepts has deepened,” and “I have participated in related discussions outside the class.” Higher scores reflect higher perceived learning performance. The Cronbach’s alpha coefficients for the three subscales were 0.895, 0.838, and 0.765, respectively, with an overall reliability of 0.930 for the adapted scale.

#### 2.2.4 Individualism

The degree of individualism was measured using a scale developed by Chen et al. (2015), consisting of 9 items. A composite individualism score was calculated by subtracting collectivism scores from individualism scores. For example, the score for the item pair “Individuals are much more important than the group” and “Individuals may not be able to survive if there is no group or country” was computed by subtracting the latter from the former. Higher scores indicate a stronger individualistic orientation. The adapted scale exhibited good internal consistency reliability (Cronbach’s *α* = 0.847).

#### 2.2.5 Intrinsic motivation

Intrinsic learning motivation was measured using a 6-item scale adapted from Fırat et al. (2018) and Lin and Wang (2021), which assessed students’ internal drive to engage in university learning activities. Sample items include: “To enrich and fulfill my inner world” and “To experience aesthetic enjoyment through learning.” Higher scores represent stronger intrinsic motivation. The adapted scale demonstrated satisfactory internal consistency (Cronbach’s *α* = 0.803).

### 2.3 Scale adaptation and measurement model validation

All instruments were adapted to the present context, following two main procedures. First, to reduce participant fatigue, redundant items were excluded any representative items were retained, with minor wording adjustments to fit the online education setting (Meng et al., 2026). Second, English-origin scales were adapted using a standard translation and back-translation procedure (Brislin, 1970) to ensure cross-cultural equivalence. Table 3 summarizes the reliability of the adapted scales. Cronbach’s α values for the overall scale ranged from 0.803 to 0.935, and from 0.765 to 0.895 for the subscales. Composite reliability (CR) values ranged from 0.859 to 0.927, all exceeding the 0.70 threshold.

**Table 3:** Reliability of the adapted scales.

Scale		Number of items	Cronbach’s *α*	CR
1. Social presence		4	0.890	0.925
2. Learning burnout	Emotional exhaustion	8	0.830	0.927
	Inappropriate learning behavior	6	0.893	
	Reduced sense of accomplishment	6	0.818	
	Total	20	0.935	
3. Individualism		9	0.847	0.880
4. Intrinsic motivation		6	0.803	0.859
5. Perceived learning performance	Perceived skill development	6	0.895	0.927
	Self-reported learning	3	0.838	
	Learning interest	3	0.765	
	Total	12	0.930	

To assess construct validity in this study, confirmatory factor analysis (CFA) was performed using Mplus. Model fit was evaluated based on commonly accepted criteria: RMSEA < 0.08, TLI > 0.90, CFI > 0.90, and a *χ*^2^/d*f* ratio below 2.5, with greater emphasis on RMSEA, TLI, and CFI (Xia and Yang, 2019). The five-factor model—including social presence, learning burnout, individualism, intrinsic motivation, and perceived learning performance—demonstrated a good fit to the data (*χ*^2^ = 781.06, d*f* = 262, *χ*^2^/d*f* = 2.98, RMSEA = 0.06, CFI = 0.93, TLI = 0.92), supporting the discriminant validity of the constructs. Moreover, following Henseler et al. (2015), we re-examined discriminant validity using the heterotrait-monotrait (HTMT) ratio of correlations. As shown in Table 4, all HTMT values were below 0.85, indicating that the constructs are empirically distinct. Taken together, the adapted scales and measurement model employed in this study demonstrated satisfactory reliability and validity.

**Table 4:** Results of discriminant validity analysis.

Variable	1	2	3	4	5
1. Social presence	—				
2. Learning burnout	0.551	—			
3. Individualism	0.269	0.382	—		
4. Intrinsic motivation	0.344	0.451	0.363	—	
5. Perceived learning performance	0.778	0.704	0.332	0.504	—

#### 2.3.1 Common method bias test

Since all data were collected through self-reported measures, the potential for common method bias was considered. Following the procedure recommended by Tang and Wen (2020), Harman’s single-factor test was conducted. The results revealed eight factors with eigenvalues greater than 1, and the first common factor accounted for 31.07% of the total variance, which was below the empirical critical value of 40% (Podsakoff et al., 2003), indicating that common method bias was not a serious concern in this study.

## 3 Results

### 3.1 Descriptive statistics and correlation analysis of main variables

Descriptive statistics, including means, standard deviations, and correlation coefficients for the main study variables, are presented in Table 5. As shown in the table, social presence was significantly positively correlated with intrinsic motivation (*r* = 0.30, *p* < 0.01) and perceived learning performance (*r* = 0.71, *p* < 0.01), and significantly negatively correlated with learning burnout (*r* = −0.48, *p* < 0.01) and individualism (*r* = −0.24, *p* < 0.01). Learning burnout was negatively correlated with intrinsic motivation (*r* = −0.37, *p* < 0.01) and perceived learning performance (*r* = −0.60, *p* < 0.01), but positively correlated with individualism (*r* = 0.34, *p* < 0.01). Individualism was negatively associated with both intrinsic motivation (*r* = −0.28, *p* < 0.01) and perceived learning performance (*r* = −0.28, *p* < 0.01). Additionally, intrinsic motivation was positively related to perceived learning performance (*r* = 0.42, *p* < 0.01). These correlations provide preliminary support for the study’s hypotheses.

**Table 5:** Results of descriptive statistics and correlation analysis.

Variable	*M*	SD	1	2	3	4	5
1. Social presence	3.56	1.39	—				
2. Learning burnout	3.76	1.05	−0.48**	—			
3. Individualism	−0.94	1.52	−0.24**	0.34**	—		
4. Intrinsic motivation	5.52	0.88	0.30**	−0.37**	−0.28**	—	
5. Perceived learning performance	4.38	1.16	0.71**	−0.60**	−0.28**	0.42**	—

** indicates *p* < 0.01.

### 3.2 Structural model and hypotheses testing

To test the proposed hypotheses, we conducted structural equation modeling (SEM) using Mplus and moderation analyses using the PROCESS macro in SPSS. First, we examined the direct and indirect effects of social presence on perceived learning performance. We hypothesized that social presence in online courses would have a direct positive effect on university students’ perceived learning performance (H1) and an indirect effect mediated by learning burnout (H2). The results indicated that social presence significantly positively predicted perceived learning performance (*b* = 0.44, SE = 0.03, *p* < 0.001), supporting H1. Further analysis revealed a significant indirect effect through learning burnout, the mediating effect was estimated at 0.08. The bootstrap 95% confidence interval for the mediating effect was [0.06, 0.11], which excluding zero, thereby supporting H2.

**Figure 2:**
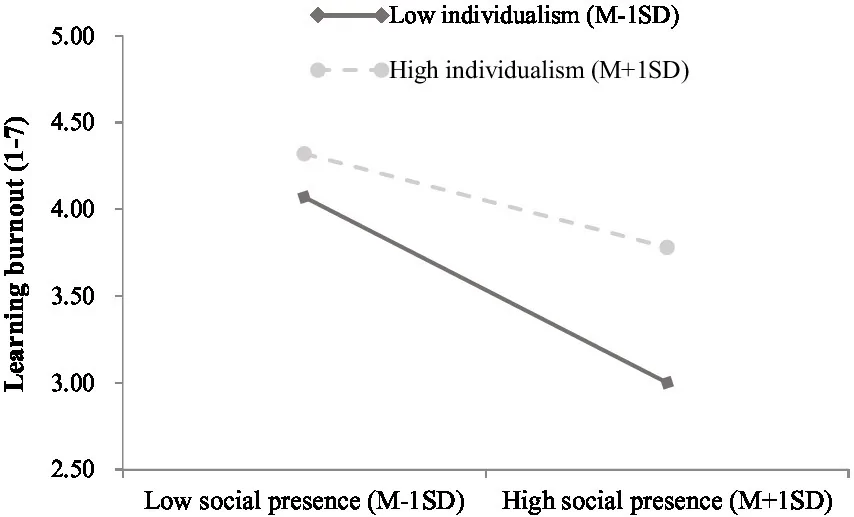
The moderating role of individualism in the relationship between social presence and learning burnout.

Next, we tested the moderation hypotheses (H3 and H5) using Hayes’ PROCESS macro (Model 1; Hayes, 2013). H3 posited that individualism moderates the relationship between social presence and learning burnout. A regression model was constructed with learning burnout as the dependent variable, social presence as the independent variable, and individualism as the moderator. Both social presence and individualism were mean-centered prior to generating the interaction term. The interaction effect was significant (*b* = 0.06, SE = 0.02, *t*(596) = 4.05, *p* < 0.001), supporting H3. To probe the interaction, we examined the conditional effects of social presence at one standard deviation above and below the mean of individualism. As illustrated in Figure 2, the effect of social presence on reducing learning burnout was stronger for students with low (–SD) individualism (*b* = −0.39, SE = 0.03, *t*(596) = −12.23, *p* < 0.001) than for those with high (+SD) individualism (*b* = −0.19, SE = 0.04, *t*(596) = −4.79, *p* < 0.001). Thus, while higher social presence consistently alleviated learning burnout, the effect was more pronounced among students low in individualism, providing further support for H3.

**Figure 3:**
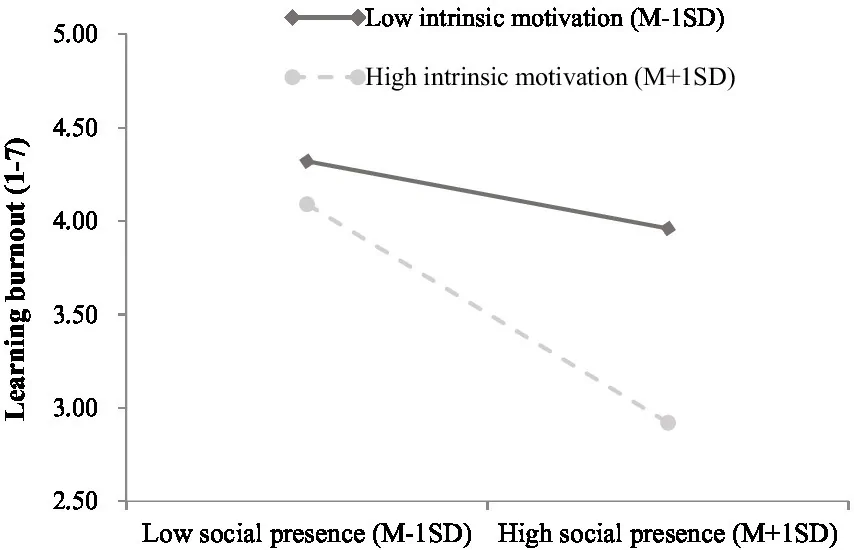
The moderating role of intrinsic motivation in the relationship between social presence and learning burnout.

Similarly, H5 proposed that intrinsic motivation moderates the relationship between social presence and learning burnout. A comparable model was constructed, with learning burnout as the dependent variable, social presence as the independent variable, and intrinsic motivation as the moderator. Both predictors were mean-centered, and their interaction term was included in the model. The interaction effect was significant (*b* = −0.17, SE = 0.03, *t*(596) = −5.62, *p* < 0.001), supporting H5. As shown in Figure 3, when intrinsic motivation was one standard deviation above the mean, the negative effect of social presence on learning burnout was stronger (*b* = −0.42, SE = 0.03, *t*(596) = −12.44, *p* < 0.001), whereas the effect was weaker but still significant when intrinsic motivation was one standard deviation below the mean (*b* = −0.13, SE = 0.04, *t*(596) = −3.14, *p* = 0.002). These results again support H5.

Finally, we examined the moderated mediation hypotheses (H4 and H6) using Mplus. H4 posited that individualism moderates the indirect effect of social presence on perceived learning performance via learning burnout. As shown in Table 6, the indirect effect was significant at both high (*b* = 0.06, 95% CI [0.03, 0.10]) and low (*b* = 0.10, 95% CI [0.07, 0.14]) levels of individualism. The difference between the two effects was also significant (*b* = −0.04, 95% CI [−0.08, −0.01]), indicating a significant moderated mediation effect and supporting H4.

**Table 6:** Moderated mediation analysis: the moderating role of individualism in the indirect effect of social presence on perceived learning performance via learning burnout.

Individualism	Effect	95% bootstrap CI	
		Lower limit	Upper limit
Low individualism (*M* − SD)	0.10	0.07	0.14
High individualism (*M* + SD)	0.06	0.03	0.10
Difference (high − low)	−0.04	−0.08	−0.01

Similarly, H6 proposed that intrinsic motivation moderates the indirect effect of social presence on perceived learning performance via learning burnout. As presented in Table 7, the conditional indirect effect was stronger when intrinsic motivation was high (*b* = 0.12, 95% CI [0.08, 0.16]) than when it was low (*b* = 0.05, 95% CI [0.02, 0.08]). The difference was statistically significant (*b* = 0.07, 95% CI [0.03, 0.12]), providing support for H6.

**Table 7:** Moderated mediation analysis: the moderating role of intrinsic motivation in the indirect effect of social presence on perceived learning performance via learning burnout.

Intrinsic motivation	Effect	95% bootstrap CI	
		Lower limit	Upper limit
Low intrinsic motivation (*M* − SD)	0.05	0.02	0.08
High intrinsic motivation (*M* + SD)	0.12	0.08	0.16
Difference (high − low)	0.07	0.03	0.12

**Figure 4:**
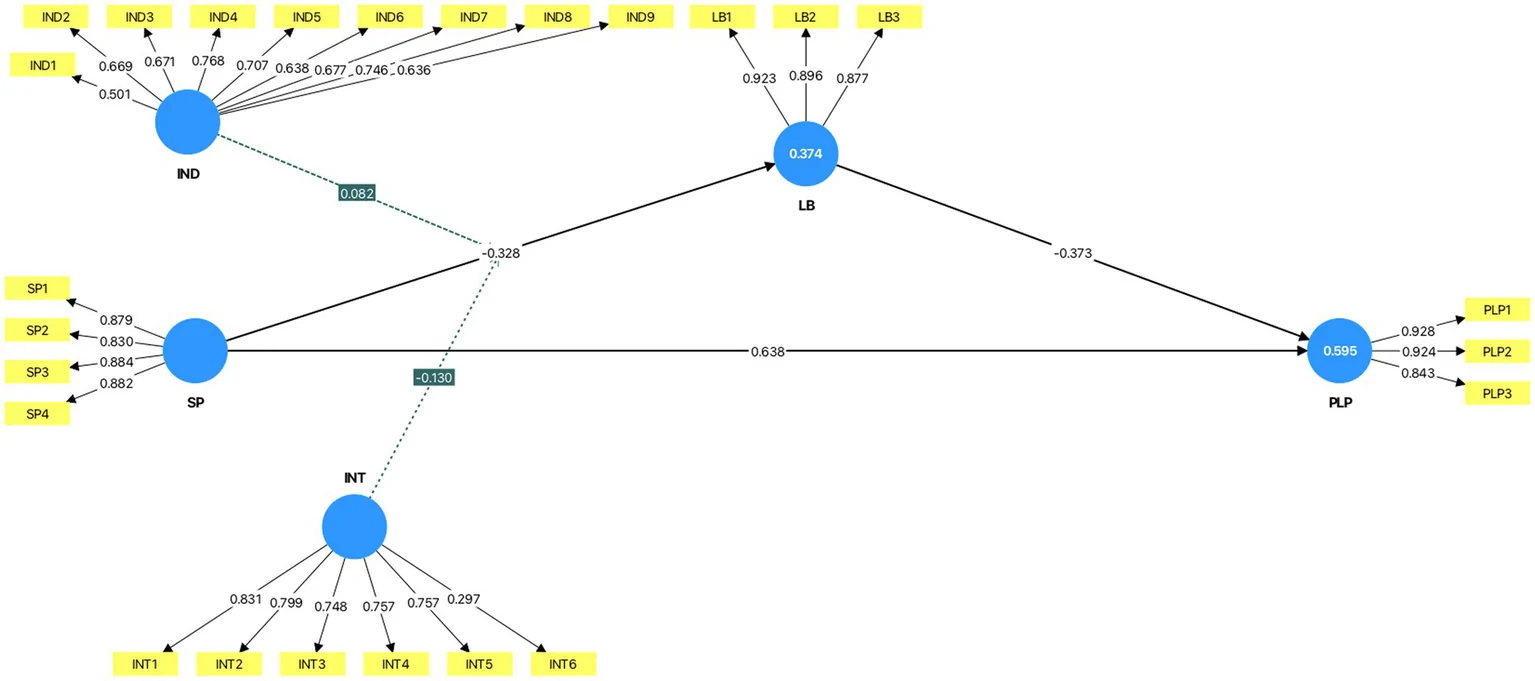
Structural equation modeling of social presence (SP), learning burnout (LB), perceived learning performance (PLP), individualism (IND), and intrinsic motivation (INT).

In sum, the results provided empirical support for all six hypotheses (H1 through H6). Moreover, to improve result visualization, we re-analyzed the structural equation model using Smart PLS software, with the results and path coefficients illustrated in Figure 4.

## 4 Discussion, conclusion, and implications

This study investigates the underlying mechanisms and boundary conditions through which social presence in online courses influences university students’ perceived learning performance. Drawing on a literature-based theoretical framework, we propose and empirically validate a model in which learning burnout mediates the positive relationship between social presence and perceived learning performance. Furthermore, we examine whether this indirect relationship is moderated by students’ individual characteristics, namely, individualism and intrinsic learning motivation.

This research makes several theoretical contributions. First, while previous research has predominantly examined the direct impact of social presence on perceived teacher support or student engagement (Miao et al., 2022; Wang, 2025), this study enriches the theoretical understanding of social presence by identifying learning burnout as a key psychological mechanism through which social presence influences perceived learning performance, thereby integrating affective dynamics into the cognitive evaluation. Second, the demonstrated mediation and moderation effects highlight the critical role of emotional and motivational processes in shaping students’ perceived learning performance. These results respond to recent calls for examining various strategies, such as interactive e-book use (Chen and Tsai, 2025), game-based learning systems (Lin et al., 2024), and goal clarity (Li et al., 2025), alongside the underlying processes to enhance students’ online learning performance. Third, by examining two individual difference variables (i.e., individualism and intrinsic motivation) as moderators, this study advances research on boundary conditions of social presence effects. Previous research has mainly focused on mediating factors, such as flow experience (Shen et al., 2024), academic buoyance and learner interactions (Yang and Lay, 2025), and academic help-seeking (Keshavarzi et al., 2024). Our findings contribute to a more nuanced understanding of how cultural values and psychological needs interact with social presence in digital learning environments. Last but not the least, our work integrates constructs from social psychology (i.e., social presence), emotion research (i.e., burnout), motivational theory (i.e., intrinsic motivation), and cross-cultural psychology (i.e., individualism) into a unified conceptual framework. This interdisciplinary model provides a more holistic perspective on university students’ experiences in online education and offers a valuable foundation for future exploration. The findings also provide practical implications for educators and instructional designers.

### 4.1 Social presence, learning burnout, and perceived learning performance

Our findings provide robust evidence that social presence significantly reduces learning burnout, which in turn enhances students’ perceived learning performance. This result is particularly meaningful in the context of online education, where the physical and emotional distance between students and teachers often leads to a sense of disconnection, reduced motivation, and increased emotional exhaustion. Compared to traditional face-to-face settings, online courses introduce technological barriers that can weaken students’ perception of teacher availability and support. These dynamics may diminish students’ engagement, arousal, and sense of learning motivation, leading to burnout and ultimately impairing learning performance. However, when social presence is effectively established through perceived teacher immediacy, responsiveness, and emotional connection, it can mitigate these adverse effects and improve performance. This finding aligns with prior meta-analytic evidence suggesting that social presence enhances students’ motivation, sense of involvement, and course satisfaction in online learning environments (Richardson et al., 2017).

Therefore, fostering a strong sense of social presence is essential in online courses such as MOOCs and other blended learning environments. Teachers can enhance social presence through visible presence in videos, immersive technologies (e.g., the metaverse), and digital game-based learning. Specific strategies include reducing the use of virtual backgrounds, simulating real classroom settings, enabling student interaction via online whiteboards or randomized questioning tools, and adopting emerging features like co-presence simulations enabled by metaverse technologies. These methods help establish a more engaging and emotionally connected learning environment, reducing feelings of isolation and learning burnout.

### 4.2 The moderating role of individualism

This study also highlights the moderating role of students’ individualism orientation. We found that students with lower levels of individualism benefited more from social presence in terms of reduced learning burnout and enhanced perceived performance. This may be because students with a lower individualism orientation tend to be more sensitive to environmental and interpersonal cues, such as teacher presence and support, and therefore experience stronger emotional and cognitive responses. In contrast, highly individualistic students are more likely to focus on the learning content itself rather than on social cues, thereby attenuating the impact of social presence. These results underscore the importance of considering cultural differences in online course design. Educators and instructional designers should tailor pedagogical approaches to fit students’ cultural profiles. For example, students from low-individualism or collectivistic cultures may benefit from interactive features, synchronous discussions, or personalized feedback that enhances their perception of teacher presence. Consistent with Hofstede’s cultural dimensions theory (Hofstede, 2001), future research could extend this line of inquiry by examining other cultural dimensions (e.g., power distance, uncertainty avoidance, and long-term orientation) and their potential impact on online education (King et al., 2018).

Thus, educators should adopt differentiated instructional strategies tailored to students’ individual characteristics. Our results show that the impact of social presence on perceived learning performance is more pronounced among students with lower individualism and higher intrinsic motivation. Teachers may use diagnostic surveys at the start of a course to identify student profiles and adjust pedagogy accordingly. For example, they could incorporate more collaborative tasks for collectivist students or facilitating autonomy-supportive learning environments for intrinsically motivated students. Techniques such as the “Think–Pair–Share” model can be used to foster student engagement, agency, and reflection.

### 4.3 The moderating role of intrinsic learning motivation

The findings also demonstrate that intrinsic learning motivation moderates the mediating pathway from social presence to perceived learning performance via learning burnout. Specifically, students with higher intrinsic motivation are more responsive to social presence cues and more likely to translate reduced burnout into improved performance. This challenges the common assumption that students with lower motivation are more reliant on external stimuli. Instead, our results suggest that intrinsically motivated students are more attuned to opportunities that support their engagement, such as the presence of the teacher. This is supported by recent findings showing that intrinsic motivation enhances students’ use of metacognitive strategies, such as self-evaluation and reflection, in online learning environments, ultimately improving task performance (Mendoza et al., 2023). According to self-determination theory (Niemiec and Ryan, 2009), social environments that satisfy students’ needs for autonomy, competence, and relatedness strengthen intrinsic motivation. Therefore, fostering social presence not only addresses emotional barriers such as burnout but also interacts with students’ motivational resources to shape positive learning experiences. This highlights the need for future research and practice to examine how to stimulate and support the development of students’ intrinsic motivation.

For practice, preventing and alleviating student burnout in online learning environments requires attention to both intrinsic and extrinsic motivational processes. We propose an interactive framework of “internal push and external pull.” Internally, courses should maintain logical flow and narrative coherence to support immersion and flow experience. Externally, teachers should leverage body language, tone variation, and expressive gestures to sustain student attention. Techniques such as unexpected cold-calling, random grouping, and dynamic pacing can add an element of surprise and engagement, helping students stay motivated and reduce burnout.

### 4.4 Limitations and future directions

Despite its contributions, this study has several limitations. First, it employed a survey design, which limits the ability to draw causal inferences. Future studies could adopt longitudinal, experimental, or mixed-method approaches (e.g., text analysis) to better explore causal relationships. In addition, due to recruitment constraints, the sample comprised a higher proportion of female participants and a relatively small number of Arts students. Thus, caution should be exercised when generalizing our findings. Future studies could include more diverse and balanced samples to further verify the robustness of the observed effects. Second, the dependent variable (i.e., perceived learning performance) was based on self-reports. Incorporating objective indicators, such as academic performance or test scores, could strengthen the robustness of the findings. Third, the average score for social presence (*M* = 3.56) suggests that students’ perceived social presence in prior online learning experiences was relatively low. This highlights the need for further research into antecedent factors that may enhance social presence. Finally, although this study confirmed the mediating role of learning burnout, future studies could explore alternative mechanisms, such as perceived psychological distance from teachers or levels of classroom engagement.

## Data Availability

The raw data supporting the conclusions of this article will be made available by the authors, without undue reservation.

## References

[ref1] Alavi M. (1994). Computer-mediated collaborative learning: an empirical evaluation. MIS Q. 18, 159–174. doi: 10.2307/249763

[ref2] Ali M. Khan A. N. Khan M. M. Butt A. S. Shah S. H. H. (2022). Mindfulness and study engagement: mediating role of psychological capital and intrinsic motivation. J. Prof. Cap. Community. 7, 144–158. doi: 10.1108/JPCC-02-2021-0013, 35579975

[ref3] Aria A. Jafari P. Behifar M. (2024). Identification of factors affecting student academic burnout in online education during the COVID-19 pandemic using grey Delphi and grey-DEMATEL techniques. Sci. Rep. 14:3989. doi: 10.1038/s41598-024-53233-7, 38368462 PMC10874453

[ref4] Barratt J. M. Duran F. (2021). Does psychological capital and social support impact engagement and burnout in online distance learning students? Internet High. Educ. 51:100821. doi: 10.1016/j.iheduc.2021.100821

[ref5] Brislin R. W. (1970). Back-translation for cross-cultural research. J. Cross-Cult. Psychol. 1, 185–216. doi: 10.1177/135910457000100301

[ref6] Chen X. Gong J. Yu B. Li S. Striley C. Yang N. . (2015). Constructs, concept mapping, and psychometric assessment of the concise scale of individualism–collectivism. Soc. Behav. Pers. 43, 667–683. doi: 10.2224/sbp.2015.43.4.667

[ref7] Chen C. C. Tsai Y. H. (2025). Effect of interactive e-book use on learning engagement, satisfaction and perceived learning. Educ. Inf. Technol. 30, 15757–15789. doi: 10.1007/s10639-025-13415-w

[ref8] Chen M. Zee M. Koomen H. M. Roorda D. L. (2019). Understanding cross-cultural differences in affective teacher-student relationships: a comparison between Dutch and Chinese primary school teachers and students. J. Sch. Psychol. 76, 89–106. doi: 10.1016/j.jsp.2019.07.011, 31759472

[ref9] Cho M. H. Heron M. L. (2015). Self-regulated learning: the role of motivation, emotion, and use of learning strategies in students’ learning experiences in a self-paced online mathematics course. Distance Educ. 36, 80–99. doi: 10.1080/01587919.2015.1019963

[ref10] Fırat M. Kılınç H. Yüzer T. V. (2018). Level of intrinsic motivation of distance education students in e-learning environments. J. Comput. Assist. Learn. 34, 63–70. doi: 10.1111/jcal.12214

[ref11] Gao W. Jiang N. Guo Q. (2023). How do virtual streamers affect purchase intention in the live streaming context? A presence perspective. J. Retail. Consum. Serv. 73:103356. doi: 10.1016/j.jretconser.2023.103356

[ref12] Greenfield P. M. Trumbull E. Keller H. Rothstein-Fisch C. Suzuki L. Quiroz B. (2006). “Cultural conceptions of learning and development,” in Handbook of Educational Psychology, eds. Winne P. Alexander P.. 2nd ed (Washington, DC: American Psychological Association), 675–692.

[ref13] Hayes A. F. (2013). An Introduction to Mediation, Moderation, and Conditional Process Analysis: A Regression-Based Approach. New York, NY: Guilford.

[ref14] Henseler J. Ringle C. M. Sarstedt M. (2015). A new criterion for assessing discriminant validity in variance-based structural equation modeling. J. Acad. Mark. Sci. 43, 115–135. doi: 10.1007/s11747-014-0403-8

[ref15] Hofstede G. (2001). Culture’s Consequences: Comparing Values, Behaviors, Institutions, and Organizations Across Nations. Thousand Oaks, CA: SAGE Publications.

[ref16] Hoogerheide V. Deijkers L. Loyens S. M. Heijltjes A. van Gog T. (2016). Gaining from explaining: learning improves from explaining to fictitious others on video, not from writing to them. Contemp. Educ. Psychol. 44-45, 95–106. doi: 10.1016/j.cedpsych.2016.02.005, 38826717

[ref17] Hoogerheide V. Renkl A. Fiorella L. Paas F. van Gog T. (2019). Enhancing example-based learning: teaching on video increases arousal and improves problem-solving performance. J. Educ. Psychol. 111, 45–56. doi: 10.1037/edu0000272

[ref18] Hsu Y.-P. Chiang D. F. Kehinde I. (2025). Transforming engineering education in the digital era: findings from a systematic review. Front. Educ. 10:1568917. doi: 10.3389/feduc.2025.1568917

[ref19] Keshavarzi F. Teo T. Heidari E. Mehrvarz M. (2024). Mediating role of academic help-seeking among students’ social networking self-efficacy and social presence in online environments. Educ. Inf. Technol. 29, 9773–9794. doi: 10.1007/s10639-023-12204-7

[ref20] Kim B. Jee S. Lee J. An S. Lee S. M. (2018). Relationships between social support and student burnout: a meta-analytic approach. Stress. Health 34, 127–134. doi: 10.1002/smi.2771, 28639354

[ref21] King R. B. McInerney D. M. Pitliya R. J. (2018). Envisioning a culturally imaginative educational psychology. Educ. Psychol. Rev. 30, 1031–1065. doi: 10.1007/s10648-018-9440-z

[ref22] Kreijns K. Kirschner P. A. Vermeulen M. (2013). Social aspects of CSCL environments: a research framework. Educ. Psychol. 48, 229–242. doi: 10.1080/00461520.2012.750225

[ref23] Li W. Wei K. Xu T. Wang J. (2025). How goal clarity affects college students’ perceived effectiveness of online self-directed learning: evidence from China. Educ. Inf. Technol. 30, 13857–13883. doi: 10.1007/s10639-024-13304-8

[ref24] Lin S. C. Lee Y. Y. (2024). Explaining the gift-giving intentions of live-streaming audiences through social presence: the perspective of interactive marketing. J. Res. Interact. Mark. 18, 945–969. doi: 10.1108/JRIM-01-2024-0030

[ref25] Lin Y. J. Wang H. C. (2021). Using virtual reality to facilitate learners’ creative self-efficacy and intrinsic motivation in an EFL classroom. Educ. Inf. Technol. 26, 4487–4505. doi: 10.1007/s10639-021-10472-9

[ref26] Lin Y. L. Wang W. T. Hsieh M. J. (2024). The effects of students’ self-efficacy, self-regulated learning strategy, perceived and actual learning effectiveness: a digital game-based learning system. Educ. Inf. Technol. 29, 22213–22245. doi: 10.1007/s10639-024-12700-4

[ref27] Lindsay A. W. (1982). Institutional performance in higher education: the efficiency dimension. Rev. Educ. Res. 52, 175–199. doi: 10.3102/00346543052002175

[ref28] Madigan D. J. Curran T. (2021). Does burnout affect academic achievement? A meta-analysis of over 100,000 students. Educ. Psychol. Rev. 33, 387–405. doi: 10.1007/s10648-020-09533-1

[ref29] Markus H. R. Kitayama S. (1991). Culture and the self: implications for cognition, emotion, and motivation. Psychol. Rev. 98, 224–253. doi: 10.1037/0033-295X.98.2.224

[ref30] Maslach C. Jackson S. E. (1981). The measurement of experienced burnout. J. Organ. Behav. 2, 99–113. doi: 10.1002/job.4030020205

[ref31] Mekler E. D. Brühlmann F. Tuch A. N. Opwis K. (2017). Towards understanding the effects of individual gamification elements on intrinsic motivation and performance. Comput. Hum. Behav. 71, 525–534. doi: 10.1016/j.chb.2015.08.048

[ref32] Mendoza N. B. Yan Z. King R. B. (2023). Supporting students’ intrinsic motivation for online learning tasks: the effect of need-supportive task instructions on motivation, self-assessment, and task performance. Comput. Educ. 193:104663. doi: 10.1016/j.compedu.2022.104663

[ref33] Meng W. Y. Kang K. W. Quan C. L. (2026). Proactive and creative personality as predictors of student engagement and innovative behavior among Chinese university students. Front. Psychol. 17:1752174. doi: 10.3389/fpsyg.2026.1752174, 41847058 PMC12990132

[ref34] Miao J. Chang J. Ma L. (2022). Teacher–student interaction, student–student interaction and social presence: their impacts on learning engagement in online learning environments. J. Genet. Psychol. 183, 514–526. doi: 10.1080/00221325.2022.2094211, 35815529

[ref35] Moreno V. Cavazotte F. Alves I. (2017). Explaining university students’ effective use of e-learning platforms. Br. J. Educ. Technol. 48, 995–1009. doi: 10.1111/bjet.12469

[ref36] Najjar N. Rouphael M. Bitar T. Hleihel W. (2025). The rise and drop of online learning: adaptability and future prospects. Front. Educ. 10:1522905. doi: 10.3389/feduc.2025.1522905

[ref37] Neumann Y. Finaly-Neumann E. Reichel A. (1990). Determinants and consequences of students’ burnout in universities. J. High. Educ. 61, 20–31. doi: 10.2307/1982032

[ref38] Niemiec C. P. Ryan R. M. (2009). Autonomy, competence, and relatedness in the classroom: applying self-determination theory to educational practice. Theory Res. Educ. 7, 133–144. doi: 10.1177/1477878509104318

[ref39] Ogara S. O. Koh C. E. Prybutok V. R. (2014). Investigating factors affecting social presence and user satisfaction with mobile instant messaging. Comput. Hum. Behav. 36, 453–459. doi: 10.1016/j.chb.2014.03.064

[ref40] Omar N. Mohamad M. M. Paimin A. N. (2015). Dimension of learning styles and students’ academic achievement. Procedia. Soc. Behav. Sci. 204, 172–182. doi: 10.1016/j.sbspro.2015.08.130

[ref41] Pap Z. Vîrgă D. Lupșa D. Crașovan M. (2021). Building more than knowledge: teacher’s support facilitates study-related well-being through intrinsic motivation. A longitudinal multi-group analysis. Learn. Individ. Differ. 88:102010. doi: 10.1016/j.lindif.2021.102010

[ref42] Park C. Kim D. G. (2020). Exploring the roles of social presence and gender difference in online learning. Decis. Sci. J. Innov. Educ. 18, 291–312. doi: 10.1111/dsji.12207

[ref43] Podsakoff P. M. MacKenzie S. B. Lee J. Y. Podsakoff N. P. (2003). Common method biases in behavioral research: a critical review of the literature and recommended remedies. J. Appl. Psychol. 88, 879–903. doi: 10.1037/0021-9010.88.5.879, 14516251

[ref44] Richardson J. C. Maeda Y. Lv J. Caskurlu S. (2017). Social presence in relation to students’ satisfaction and learning in the online environment: a meta-analysis. Comput. Hum. Behav. 71, 402–417. doi: 10.1016/j.chb.2017.02.001

[ref45] Romano L. Angelini G. Consiglio P. Fiorilli C. (2021). The effect of students’ perception of teachers’ emotional support on school burnout dimensions: longitudinal findings. Int. J. Environ. Res. Public Health 18:1922. doi: 10.3390/ijerph18041922, 33671176 PMC7922948

[ref46] Ryan R. M. Deci E. L. (2000). Self-determination theory and the facilitation of intrinsic motivation, social development, and well-being. Am. Psychol. 55, 68–78. doi: 10.1037/0003-066X.55.1.68, 11392867

[ref47] Shen Q. Gao B. Lu J. Ge Q. Yan X. Chen B. (2024). Overcoming the “screen dilemma”: the psychological mechanisms of social presence promoting college students’ online learning engagement. Educ. Inf. Technol. 29, 21095–21114. doi: 10.1007/s10639-024-12706-y

[ref48] Short J. Williams E. Christie B. (1976). The Social Psychology of Telecommunications. Hoboken, NJ: Wiley.

[ref49] Singh A. Ishrat A. (2025). The role of social support in enhancing self-efficacy and learning satisfaction in online education among secondary school students. On. Horizon. Internat. J. Learn. Futur. 33, 320–337. doi: 10.1108/OTH-03-2025-0033

[ref50] Sun A. Chen X. (2016). Online education and its effective practice: a research review. J. Inf. Technol. Educ. Res. 15, 157–190. doi: 10.28945/3502

[ref51] Tan K. L. Cyril Eze U. Sun Y. (2025). I did my part! How can I further minimize emerging adult learners’ burnout in an online learning environment? Educ. Stud. 51, 58–80. doi: 10.1080/03055698.2022.2119370

[ref52] Tang D. Wen Z. (2020). Statistical approaches for testing common method bias: problems and suggestions. J. Psychol. Sci. 43, 215–223.

[ref53] Tang L. Zhang F. Yin R. Fan Z. (2021). Effect of interventions on learning burnout: a systematic review and meta-analysis. Front. Psychol. 12:645662. doi: 10.3389/fpsyg.2021.645662, 33716914 PMC7952745

[ref54] Teoh H. Y. Serang D. P. Lim C. C. (1999). Individualism-collectivism cultural differences affecting perceptions of unethical practices: some evidence from Australian and Indonesian accounting students. Teach. Bus. Ethics 3, 137–153. doi: 10.1023/A:1009832018849

[ref55] Wan Z. Wang Y. Haggerty N. (2008). Why people benefit from e-learning differently: the effects of psychological processes on e-learning outcomes. Inf. Manag. 45, 513–521. doi: 10.1016/j.im.2008.08.003

[ref56] Wang Y. (2025). The role of teachers’ social support in students’ emotional and cognitive presences in online learning. Distance Educ. 46, 209–224. doi: 10.1080/01587919.2024.2346250

[ref57] Wang J. Antonenko P. D. (2017). Instructor presence in instructional video: effects on visual attention, recall, and perceived learning. Comput. Hum. Behav. 71, 79–89. doi: 10.1016/j.chb.2017.01.049

[ref58] Wang Q. Li X. Yan X. (2025). When the mindful ones experience flow: a moderated-mediation model of purchase intention in live commerce. Inf. Technol. People 39, 635–667. doi: 10.1108/ITP-04-2023-0377

[ref59] Wang Y. Stein D. Shen S. (2021). Students’ and teachers’ perceived teaching presence in online courses. Distance Educ. 42, 373–390. doi: 10.1080/01587919.2021.1956304

[ref60] Watt H. M. Carmichael C. Callingham R. (2017). Students’ engagement profiles in mathematics according to learning environment dimensions: developing an evidence base for best practice in mathematics education. Sch. Psychol. Int. 38, 166–183. doi: 10.1177/0143034316688373

[ref61] Wei H. Dorn A. Hutto H. Webb Corbett R. Haberstroh A. Larson K. (2021). Impacts of nursing student burnout on psychological well-being and academic achievement. J. Nurs. Educ. 60, 369–376. doi: 10.3928/01484834-20210616-02, 34232812

[ref62] Weidlich J. Yau J. Kreijns K. (2024). Social presence and psychological distance: a construal level account for online distance learning. Educ. Inf. Technol. 29, 401–423. doi: 10.1007/s10639-023-12289-0

[ref63] Wu Y. Dai X. Wen Z. Cui H. (2010). Development of the adolescent learning burnout scale. Chin. J. Clin. Psychol. 18, 152–154.

[ref64] Xia Y. Yang Y. (2019). RMSEA, CFI, and TLI in structural equation modeling with ordered categorical data: the story they tell depends on the estimation methods. Behav. Res. Methods 51, 409–428. doi: 10.3758/s13428-018-1055-2, 29869222

[ref65] Xiong Y. Li H. Kornhaber M. L. Suen H. K. Pursel B. Goins D. D. (2015). Examining the relations among student motivation, engagement, and retention in a MOOC: a structural equation modeling approach. Glob. Educ. Rev. 2, 23–33.

[ref66] Yang Y. Lay Y. F. (2025). Academic buoyancy and learner interactions as mediators of deep learning in blended learning contexts: the role of teaching, social, and cognitive presence. Educ. Inf. Technol. 30, 6261–6286. doi: 10.1007/s10639-024-13066-3

[ref67] Yildirim Y. Alptekin G. Altinpulluk H. Kilinc H. Yumurtaci O. (2024). Loneliness and intrinsic motivation levels of distance learners in virtual environments. Educ. Inf. Technol. 29, 11023–11041. doi: 10.1007/s10639-023-12274-7

[ref68] Younas M. Alenezi A. T. El-Dakhs D. A. S. (2026). Digital literacy and privacy concerns as determinants of next-generation technology adoption in higher education: an extended UTAUT2 perspective. Front. Educ. 11:1814830. doi: 10.3389/feduc.2026.1814830

[ref69] Zhang Y. Tian Y. Yao L. Duan C. Sun X. Niu G. (2022). Individual differences matter in the effect of teaching presence on perceived learning: from the social cognitive perspective of self-regulated learning. Comput. Educ. 179:104427. doi: 10.1016/j.compedu.2021.104427

